# Endophytic Bacterium *Serratia plymuthica* From Chinese Leek Suppressed Apple Ring Rot on Postharvest Apple Fruit

**DOI:** 10.3389/fmicb.2021.802887

**Published:** 2022-03-03

**Authors:** Meng Sun, Junping Liu, Jinghui Li, Yonghong Huang

**Affiliations:** ^1^College of Horticulture, Qingdao Agricultural University, Qingdao, China; ^2^Laboratory of Quality and Safety Risk Assessment for Fruit (Qingdao), Ministry of Agriculture and Rural Affairs, Qingdao, China; ^3^National Technology Centre for Whole Process Quality Control of FSEN Horticultural Products (Qingdao), Qingdao, China; ^4^Qingdao Key Laboratory of Modern Agriculture Quality and Safety Engineering, Qingdao, China

**Keywords:** *Serratia plymuthica*, *Botryosphaeria dothidea*, fruit quality, biocontrol, RNA-seq

## Abstract

Apple ring rot caused by *Botryosphaeria dothidea* is an economically significant plant disease that spreads across the apple production areas in China. The pathogen infects apple fruits during the growing season and results in postharvest fruits rot during storage, which brings about a huge loss to plant growers. The study demonstrated that an endophytic bacterium *Serratia plymuthica* isolated from Chinese leek (*Allium tuberosum*) significantly suppressed the mycelial growth, severely damaging the typical morphology of *B. dothidea*, and exerted a high inhibition of 84.64% against apple ring rot on postharvest apple fruit. Furthermore, *S. plymuthica* significantly reduced the titratable acidity (TA) content, enhanced the soluble sugar (SS) content, vitamin C content, and SS/TA ratio, and maintained the firmness of the fruits. Furthermore, comparing the transcriptomes of the control and the *S. plymuthica* treated mycelia revealed that *S. plymuthica* significantly altered the expressions of genes related to membrane (GO:0016020), catalytic activity (GO:0003824), oxidation-reduction process (GO:0055114), and metabolism pathways, including tyrosine metabolism (ko00280), glycolysis/gluconeogenesis (ko00010), and glycerolipid metabolism (ko00561). The present study provided a possible way to control apple ring rot on postharvest fruit and a solid foundation for further exploring the underlying molecular mechanism.

## Introduction

Apple ring rot caused by *Botryosphaeria dothidea* is one of the devastating diseases spreading over China’s central apple production region (Dong et al., 2021). *B. dothidea* infects branches, trunk, or fruits *via* lenticel or wound and results in warts in shoots and trunk or lesions in ripened fruits. As the disease progresses, it causes the infected trunk and shoots to die or fruits to rot (Bai et al., 2015). According to a previous report in 2009, the average occurrence of the apple ring rot is as high as 77.6% in 88 apple orchards across seven main apple production areas in China (Guo et al., 2009). Thus, the disease has been seriously threatening the healthy development of apple production in China. In addition, besides apple trees, *B. dothidea* can infect other essential fruit trees, such as pear (*Pyrus bretschneideri* Rehd.) (Sun et al., 2020), pomegranate (*Punica granatum* L.) (Gu et al., 2020), apricot (*Prunus armeniaca* L.) (Huang et al., 2019b), kiwifruit (*Actinidia chinensis*) (Wang et al., 2021), sweet cherry ( *Prunus avium* L.) (Zhang et al., 2019), and mulberry (*Morus alba* L.) (Huang et al., 2019a). Therefore, it is particularly important to develop ways to prevent and control the disease caused by *B. dothidea*.

Presently, chemical fungicides are still the most widely used methods to control apple ring rot, but it has hazards such as pollution to the environment and harm to human health and animals. Researchers have found that adopting antagonistic microbes is an efficient, safer, and environment-friendly way to control the disease. Previous studies show that *Bacillus amyloliquefaciens* PG12 (Chen et al., 2016), *Bacillus subtilis* 9407 (Fan et al., 2017), *Streptomyces rochei* A-1 (Zhang et al., 2016), *Streptomyces lavendulae* Xjy (Gao et al., 2016), *Paenibacillus polymyxa* APEC136 (Kim et al., 2016), *Bacillus atrophaeus* J-1 (Mu et al., 2020), and *Meyerozyma guilliermondii* Y-1 (Huang et al., 2021) efficiently suppress *B. dothidea* growth and reduce apple ring rot occurrence on fruits or apple trees.

Our previous study shows Chinese leek (*Allium tuberosum*) extract significantly suppresses the apple ring rot on apple shoots and fruits (Zhao et al., 2017) and reduces the occurrence of banana fusarium wilt caused by *Fusarium oxysporum* f. sp. *cubense* race 4 (*Foc*4) (Huang et al., 2012). Later, we isolated an endophytic bacterium from Chinese leek, identified as *S. plymuthica*, using 16 sRNA (Müller et al., 2013; Adam et al., 2016). Previous studies show that *S. plymuthica* has potent antifungal activity against several plant pathogens. *S. plymuthica* exerts striking inhibition against root rot on cucumber plant caused by *Pythium ultimum* (Benhamou et al., 2000). *S. plymuthica* strains IC1270 and IC14 control *Penicillium digitatum* (green mold) or *Penicillium italicum* (blue mold) on orange (Meziane et al., 2006). *S. plymuthica* A30 reduces blackleg development by 58.5% and transmission to tuber progeny as latent infection by 47-75% (Hadizadeh et al., 2019). *S. plymuthica* Sneb2001 shows a high lethal effect on the second-stage juveniles of *Meloidogyne incognita* (Zhao et al., 2021). However, to our knowledge, there are no reports concerning the effects of *S. plymuthica* on *B. dothidea*. Therefore, in the present study, we attempt to determine whether the endophytic bacterium *S. plymuthica* inhibits the apple ring rot on postharvest fruits caused by *B. dothidea* and preliminarily explore the underlying molecular mechanism, trying to provide a possible way to control apple ring rot.

## Materials and Methods

### Experimental Materials

The apple fruit (*Malus domestica* Borkh. cv. Red Fuji) used in the experiments were purchased in the local supermarkets. The fruit with uniform sizes, no disease spots, and no mechanical damages was selected for the experiments. The *S. plymuthica* and the *B. dothidea* were kept on nutrient agar (NA) medium and potato dextrose agar (PDA) medium in the laboratory, respectively.

### Determination of the Inhibition of *Serratia plymuthica* on the Growth of *Botryosphaeria dothidea*

PDA medium (20 ml) was poured into a 9-cm-diameter Petri dish. One fungal mycelium disc 0.5 cm in diameter was inoculated at 1/3 of Petri dish diameter. *S. plymuthica* culture (50 μl) (OD_600_ = 0.6) was added at 2/3 of Petri dish diameter. Sterilized water (50 μl) was used as untreated control. All the Petri dishes were inverted and incubated at 28°C for 3 days in the dark. The fungus mycelium diameters were measured every day to evaluate the inhibition of *S. plymuthica* on *B. dothidea* growth. The experiments were repeated three times, and five replicates were included for each sample in each experiment.

### Observation of *Botryosphaeria dothidea* Mycelial Morphology Treated With *Serratia plymuthica*

A layer of cellophane was laid on a newly prepared PDA medium. One fungal mycelium disc (0.5 cm in diameter) was inoculated on the cellophane. *S. plymuthica* culture (50 μl) (OD_600_ = 0.6) was added onto a small piece of filter paper (1 × 1 cm), 1 cm away from the mycelium disc, over the cellophane. The fungal mycelium disc without *S. plymuthica* was used as an untreated control. The Petri dishes were inverted and incubated at 28°C in the dark. Twenty-four hours later, the cellophane with new mycelium was taken out from the Petri dishes, cut into 1 cm × 1 cm squares, and was observed under a microscope (EVOS Auto2, Thermo Fisher Scientific, United States).

### The Inhibition of *Serratia plymuthica* Against Apple Ring Rot on Fruits Caused by *Botryosphaeria dothidea*

The apple fruits with uniform size, healthy, no mechanical injury were selected as experimental materials. After the fruit was sterilized with 70% ethanol, a tiny cavity (0.5 cm in diameter and 0.5 cm in depth) was made at the equator of the sterilized fruits using a sterilized puncher. Then, 50 μl *S. plymuthica* (OD_600_ = 0.6) was added into the small cavity. Then, 1 h later, one fungal mycelium disc (0.5 cm in diameter) was inoculated into the small cavity (*S. plymuthica* treated). The nutrient broth (NB) medium was used as a control. All the treated and the control fruits were placed in an incubator at 28°C for 5 days. The disease symptoms were recorded every day to evaluate the inhibition of *S. plymuthica* against apple ring rot caused by *B. dothidea*. The experiments were repeated three times, and 12 replicates were included for each sample in each experiment.

### Effects of *Serratia plymuthica* on the Fruit Qualities

To determine whether *S. plymuthica* affected apple fruit quality, we designed four treatments in the experiment. (1) The apple fruits were soaked in PDB medium for 15 min (CK). (2) The apple fruits were soaked in *B. dothidea* culture for 15 min (Bd). (3) The apple fruits were soaked in *S. plymuthica* culture (OD_600_ = 0.6) for 15 min (Sp). (4) The apple fruits were firstly soaked in *S. plymuthica* culture (OD_600_ = 0.6) culture for 15 min, and then they were aired at room for 1 h followed by soaking in *B. dothidea* culture for 15 min (Sp+Bd). All the fruits were set in an incubator at 28°C. Then, at 1, 3, 5, 7, and 9 days, fruits were sampled and peeled. Fruit firmness was measured at the fruit’s equator with the FHM-5 fruit hardness tester (Takemura Electric Works Ltd., Tokyo, Japan). The pulp at the equator of the fruit was sampled to determine other internal quality indexes, including total soluble solid (TSS), soluble sugar (SS), titratable acidity (TA), vitamin C (VC), total soluble solid/titrable acidity (TSS/TA), and soluble sugar/titrable acidity (SS/TA). TSS content was determined using a PAL-1 type sugar concentration detector (ATAGO, Japan). SS content was determined using the anthrone colorimetric method. The TA content was determined by NaOH titration. VC content was determined by 2, 6-dichloroindophenol colorimetric (Yang et al., 2020). Three replicates were included for each sample in each sampling time point. The area-under-curve (AUC) of the internal quality indexes was calculated as the formula (Huang et al., 2012).


(1) $$\text{AUC}=\sum_{i=1}^{n-1}\left[\frac{X_{i+1}+X_{i}}{2}\right]\left(t_{i+1}-t_{i}\right)$$

*X* is fruit internal quality indexes. *n* is the number of evaluations, and (*t*_*i*_-_1_-*t*_*i*_) is the time interval (days) between two consecutive evaluations.

### RNA-Seq Analysis

A layer of sterilized cellophane was placed on the newly prepared PDA medium, on the center of which a mycelium disc (0.5 cm in diameter) of *B. dothidea* was inoculated. The Petri dish was sealed, inverted, and incubated at 28°C in the dark for 2 days. *S. plymuthica* culture (300 μl) (OD_600_ = 0.6) was sprayed on the mycelia. Sterilized water (300 μl) was used as control. The Petri dishes continued to be incubated in the same condition. Finally, 6 and 12 h later, the *S. plymuthica*-treated and the control mycelia were sampled for RNA-seq analysis and qRT-PCR verification.

Total RNA was extracted from *B. dothidea* mycelia using RNAprep Pure Plant Kit (Polysaccharides and Polyphenolics-rich) (Tiangen Biotech, Beijing). The quality and integrity of RNA samples were determined using a NanoDrop-2000 Spectrophotometer (Thermo Fisher Scientific, United States), agarose gel electrophoresis, and an Agilent Bioanalyzer 2100 bioanalyzer (Agilent Technologies, United States). The mRNA was isolated and fragmented into short segments (200–300 nt). The first-strand cDNA was generated by using the random hexamer-primed reverse transcription. Subsequently, the second strand was synthesized using RNase H and DNA polymerase I. Double-strand cDNA was purified and end-repaired, added with poly (A) tail, and ligated to sequencing linkers. The fragments were purified and then enriched by using PCR amplification to construct the final cDNA library. The library quality was detected with the Agilent Bioanalyzer 2100 bioanalyzer (Agilent Technologies, United States). The cDNA library was then sequenced *via* Illumina HiSeq™ 2500. The data were deposited at the National Center for Biotechnology Information (accession PRJNA727902).

After sequencing, the low-quality reads and adapter sequences were removed from the raw data using Cutadapt (v2.10) (Martin, 2000). Then, the high-quality clean data were mapped to the *B. dothidea* genome (ASM1150312v2) with TopHat 2.1.1 (Trapnell et al., 2009). Gene expression levels were quantified based on HTSeq (Anders et al., 2015), the values of which were normalized by FPKM (Fragments Per Kilo bases per Million fragments) method. The differentially expressed genes (DEGs) analysis was performed by DESeq (Wang et al., 2010) with a threshold of | log2foldchang| > 1 and *P* < 0.05. The DEGs were functionally annotated by mapping to the Gene Ontology (GO) (Ashburner et al., 2000), Kyoto Encyclopedia of Genes and Genomes (KEGG) (Kanehisa et al., 2016). We defined GO terms and KEGG pathways with a *q*-value < 0.05 as significantly enriched.

### Quantitative Real-Time PCR Verification

DEGs were randomly selected for quantitative real-time PCR (qRT-PCR) to validate the quality of the sequencing data. The selected genes were extracted from the *B. dothidea* genome (ASM1150312v2), and the special primers were designed at the Primer Premier 5.0 software (Table 1). The cDNA was synthesized using the HiScript^®^ lll RT SuperMix for qPCR (+gDNA wiper) reverse transcription kit (Vazyme Biotech, Nanjing, CN). According to the instructions of the ChamQ™ SYBR Color qPCR Master Mix kit (Vazyme Biotech, Nanjing, CN), the expressions of selected genes were analyzed by ABI7500 thermal cycler (Applied Biosystems, CA). The total reaction system was 10 μl, including 5 μl of 2 × ChamQ SYBR Color qPCR Master Mix, 0.2 μl of each primer, 0.2 μl of the 50 × ROX Reference Dye I, 1 μl of cDNA, and 3.4 μl of the ddH_2_O. The reaction conditions were following: 94°C for 5 min, followed by 30 cycles of 94°C for 30 s and 60°C for 30 s, then 72°C for 30 s, and a final extension at 72°C for 10 min. Actin was used as the internal reference gene, and the relative expression was calculated by 2^–ΔΔCT^ method (Livak and Schmittgen, 2001). Three biological replicates were set up in the experiment.

**TABLE 1:** The qRT-PCR primers using in the study.

Gene	Forward primer sequence	Reverse primer sequence
BOTSDO06531	5′-ATGACCACACCACACTGCT-3′	5′-CTCCTTCTTCTGTTGCGACG-3′
BOTSDO00320	5′-AACAAACCGCTCGCTCAATC-3′	5′-CTGTTCTCCTCAATGCCGTG-3′
BOTSDO08455	5′-CGACGTCCTCCTCTACTTCC-3′	5′-GGCGTCTCGGTCCTACTG-3′
BOTSDO10612	5′-CAATGAGTCTCACCGCCATG-3′	5′-ACACAATTGACCGCGTTTCA-3′
BOTSDO00589	5′-TGCAATTCCTCACCCTCACC-3′	5′-GTTGTTGTGGCAGACGTAGG-3′
BOTSDO08313	5′-GCTTCAAGGCTTTCGAGGAC-3′	5′-TCGTACTGGTCCTTGTCACC-3′
BOTSDO02077	5′-CAAACCCTCACCATCCTCCT-3′	5′-CTGGATCAGGAAGGACAGCA-3′
BOTSDO04628	5′-CACTCCATCCTCTTCCTCCC-3′	5′-CGAGTTATCCCGCGAAATGG-3′
BOTSDO06884	5′-AAGAAGTTCCTCCAGCACGA-3′	5′-CATCCCACATAGCCGCATTC-3′
BOTSDO11898	5′-AATACGCCGCCAACATCTTC-3′	5′-CAGGTTGGAGACTAGGCGAA-3′
BOTSDO07469	5′-CTTCACCACCCTCCTCACC-3′	5′-TTCATGACGAAGCGGTTGAG-3′
BOTSDO08150	5′-GGTCTCCATCTTCCTTCGCT-3′	5′-CCAGATCAAGGAGGTGACGA-3′
BOTSDO13437	5′-CATTTCCTCCCTCAACGCAG-3′	5′-TCCCCTCTCCTTACTGCTCT-3′
BOTSDO01196	5′-GCTAGAGTCGAACGGGTACA-3′	5′-CGGGTCGACATCAATAGGGA-3′
BOTSDO11589	5′-CAAGGCTGCCACTGGAAAAT-3′	5′-CACGTCCAAAACAACCGGAT-3′
Actin	5′-GTTCAGACCGCCCTTTGCT-3′	5′-AGCCTTGCGACGGAACATA-3′

### Statistical Analysis

Experimental data were analyzed using standard analysis of variance (ANOVA) followed by least significant difference tests (*P* < 0.05) using the software statistical analytical system (SAS 9.0). Standard errors were calculated for all mean values.

## Results

### *Serratia plymuthica* Significantly Inhibited the Mycelial Growth of *Botryosphaeria dothidea*

**FIGURE 1:**
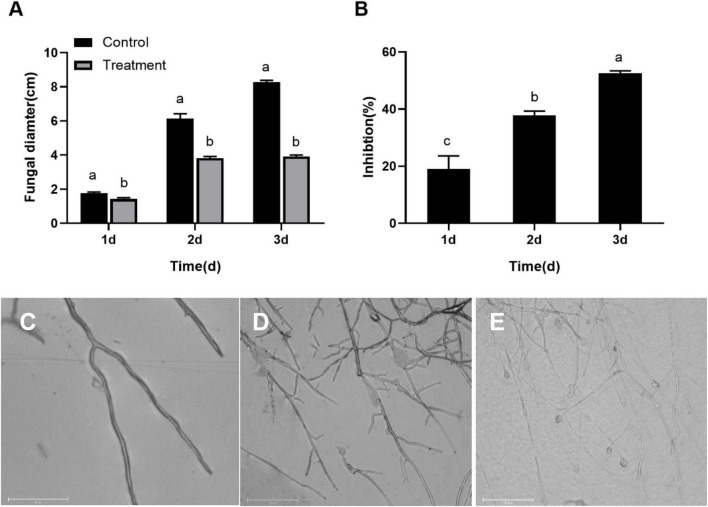
*Serratia plymuthica* significantly inhibits the mycelial growth of *Botryosphaeria dothidea* **(A)**. The inhibition increases with the increase of the treatment time **(B)**. Compared to the untreated control **(C)**, *Serratia plymuthica* damages the mycelial morphology of *Botryosphaeria dothidea* **(D,E)**. Different lowercase letters indicate significant differences between treatments or different times (*P* < 0.05).

The *B. dothidea* mycelium discs inoculated on the PDA medium without *S. plymuthica* (control) began to grow on the first day and quickly expanded later. The mycelia covered the whole surface of the PDA medium after 3 day. But the *B. dothidea* mycelium discs treated with *S. plymuthica* grew more slowly than the control. Statistics showed the mycelium diameters treated with *S. plymuthica* were 1.42, 3.83, and 3.93 cm at 1, 2, and 3 days, respectively, significantly smaller than those of the control. Compared to the control, *S. plymuthica* suppressed the growth of *B. dothidea* by 19.01% (*P* = 0.0086), 37.81% (*P* < 0.0001), and 52.50% (*P* < 0.0001) at 1, 2, and 3 days, respectively (Figure 1A). Furthermore, the inhibition increased with the increase of the treatment time (*P* < 0.0001) (Figure 1B).

### *Serratia plymuthica* Deformed the Typical Morphology of *Botryosphaeria dothidea* Mycelia

To identify whether *S. plymuthica* damaged the typical morphology of *B. dothidea* mycelia, we compared the control (Figure 1C) and the *S. plymuthica-*treated mycelia (Figures 1D,E). Under the microscope, the control mycelia had a smooth surface, with uniform sizes and fewer branches. But the *S. plymuthica*-treated mycelia showed abnormal morphology and became uneven in thickness. They showed more forks and swelled on the top end. In addition, some mycelia were so seriously damaged, resulting in a burst with the contents spilling out.

### *Serratia plymuthica* Inhibited the Apple Ring Rot Occurrence on Fruits Caused by *Botryosphaeria dothidea*

**FIGURE 2:**
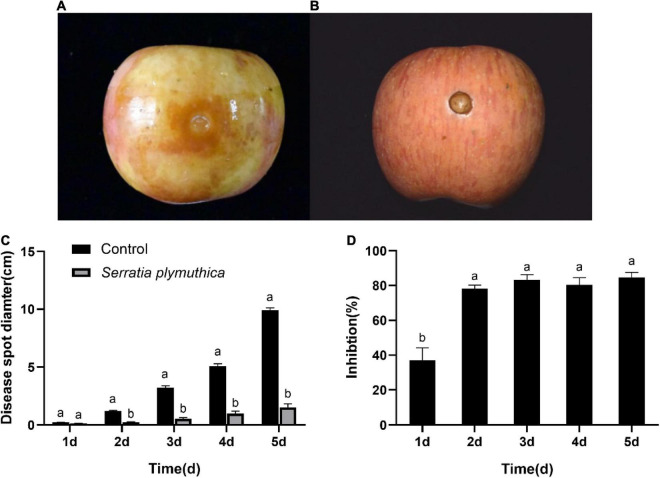
The disease symptoms on the apple fruit inoculated with *Botryosphaeria dothidea*. The disease develops quickly on control fruits, and apple fruit rots 5 days later **(A)**. But the *Serratia plymuthica*-treated apple only shows slight disease symptoms **(B)**. *Serratia plymuthica* significantly reduces the disease spot diameter **(C)** and inhibits apple ring rot on postharvest fruits **(D)** caused by *Botryosphaeria dothidea*. Different lowercase letters indicate significant differences between treatments or different times (*P* < 0.05).

All the *S. plymuthica*-treated and the untreated apple fruits showed slight disease symptoms at the inoculation sites 1 day later. But the disease spread at varying rates in the next few days. The disease spot diameter on the control fruit rapidly increased, almost as large as the height of the apple fruits 5 days later (Figure 2A). However, the disease symptom on the *S. plymuthica*-treated apple fruits developed slowly, and the disease spot diameter was smaller than the control (Figure 2B). Statistics analysis showed *S. plymuthica* significantly reduced the disease spot diameters on apple fruits compared to the control at 2 days (*P* < 0.0001), 3 days (*P* = 0.0028), 4 days (*P* = 0.0025), and 5 days (*P* = 0.0002) (Figure 2C), which showed *S. plymuthica* exhibited an efficient inhibition against the apple ring rot on fruits. In addition, the inhibition significantly increased with the duration of *S. plymuthica* treatment (*P* = 0.0016). On the first day, the inhibition was 37.04%. It reached 78.01% on the second day, then climbed as high as 84.64% on the subsequent days (Figure 2D).

### *Serratia plymuthica* Enhanced the Fruit Quality Indexes of Apple Fruit

**FIGURE 3:**
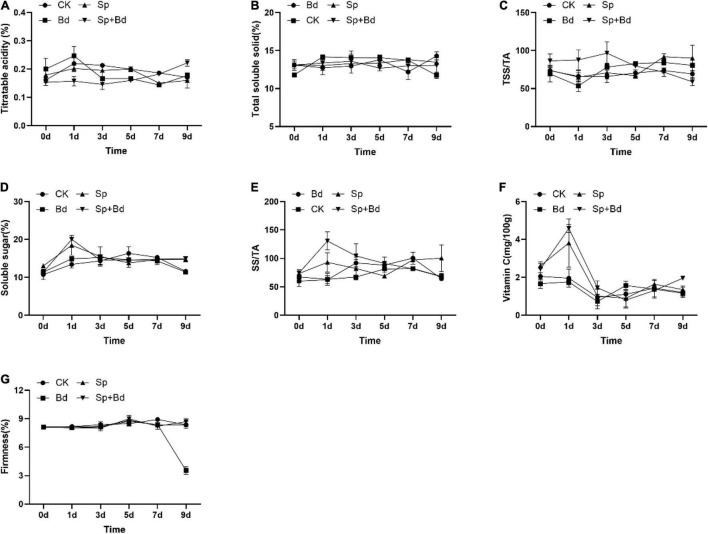
The dynamic changes of fruit quality indexes including titratable acidity **(A)**, total soluble solid **(B)**, TSS/TA **(C)**, SS **(D)**, SS/TA **(E)**, vitamin C **(F)**, and firmness **(G)** in the different treatment fruits.

**FIGURE 4:**
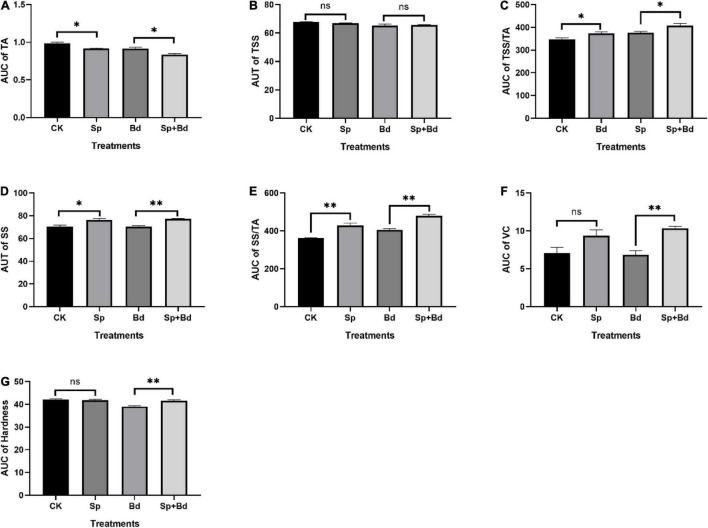
The area-under-curve (AUC) of fruit quality indexes including titratable acidity **(A)**, total soluble solid **(B)**, TSS/TA **(C)**, SS **(D)**, SS/TA **(E)**, vitamin C **(F)**, and firmness **(G)** in the different treatment fruits. “*” means *P* < 0.05, “^**^” means *P* < 0.01, “ns” means *P* < 0.05.

The seven indexes were determined to test the effects of *S. plymuthica* on the fruit quality. The results showed that all the indexes were variable during the experiment period, exhibiting an up- and down trend with the extension of the experiment time (Figure 3). The AUC showed whether the apple fruit was inoculated with *B. dothidea*, *S. plymuthica* significantly reduced TA content, enhanced SS content, TSS/TA ratio, and SS/TA ratio in apple fruits (Figure 4). Compared to the control (CK), TA content in *S. plymuthica*-treated apple fruit (Sp) was reduced by 6.91% (*P* = 0.0226), and the SS content, TSS/TA ratio, and SS/TA ratio were enhanced by 8.27% (*P* = 0.0307), 8.28% (*P* = 0.0149), and 18.54% (*P* = 0.0074), respectively. In addition, compared to the control (Bd), TA content in *S. plymuthica*-treated apple fruit (Sp+Bd) was reduced by 8.69% (*P* = 0.0329), and the SS content, TSS/TA ratio, and SS/TA were enhanced by 9.39% (*P* = 0.0036), 9.20% (*P* = 0.0352), and 18.13% (*P* = 0.0030), respectively. Moreover, *S. plymuthica* also significantly improved VC content and maintained firmness when the apple fruit was inoculated with *B. dothidea.* The VC content and firmness in *S. plymuthica*-treated fruit (Sp+Bd) were 50.82% (*P* = 0.0048) and 6.55% (*P* = 0.0085) higher than that of control (Bd).

### RNA-Seq Analysis and Differential Expression Gene Screening

**FIGURE 5:**
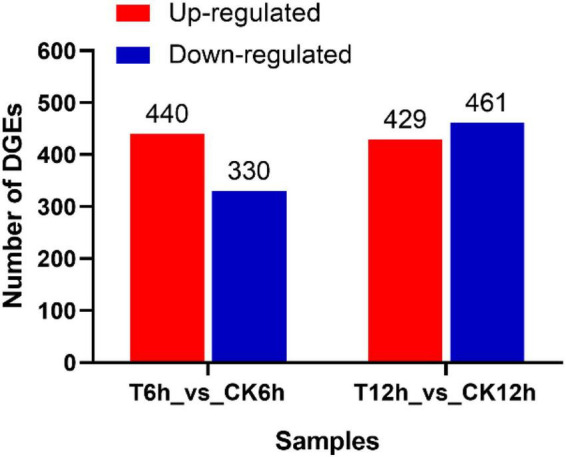
Differentially expressed genes screening in *Botryosphaeria dothidea* treated by *Serratia plymuthica.*

After the raw data (35.02 Gb) were filtered to remove low-quality reads, the resulting high-quality clean data (26.97 Gb) were aligned to the *B. dothidea* reference genome (ASM1150312v2). Then, DEGs in the *S. plymuthica*-treated and the untreated control *B. dothidea* mycelium were screened by DESeq according to the screening criteria of | LogFoldChange| > 1 and P < 0.05 (Supplementary Table 1). At 6 h, 770 DEGs were obtained from comparison of T6h and CK6h samples, 440 (57.1%) of which were upregulated, and 330 (42.9%) downregulated. At 12 h, the DEGs were up to 890 (T12h_vs_CK12h), 429 (48.2%) of which were upregulated and 461 (51.8%) downregulated (Figure 5).

### Gene Ontology Analysis

**FIGURE 6:**
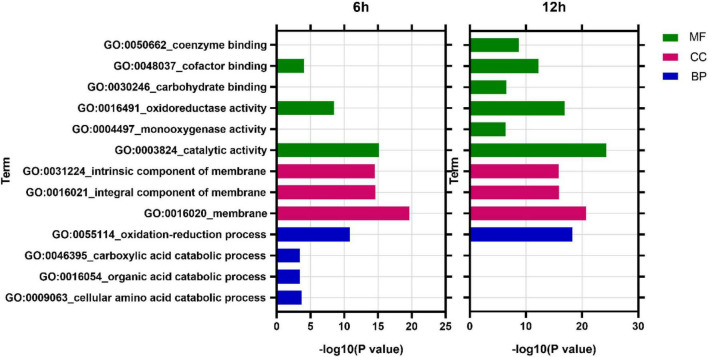
GO enriched analysis of differentially expressed genes. MF, Molecular function; CC, cellular component; BP, biological process.

GO analysis revealed that at 6 h, DEGs were enriched into 838 items, among which 490 (58.5%), 43 (5.1%), and 305 (36.4%) belonged to biological process (BP), cellular component (CC), and molecular function (MF), respectively. At 12 h, DEGs were enriched into 901 items, of which 519 (57.6%), 51 (5.7%), and 331 (36.7%) belonged to BP, CC, and MF, respectively (Supplementary Table 2). In addition, analysis of the most enriched 10 items in each time point found that membrane (GO:0016020, CC), catalytic activity (GO:0003824, MF), and oxidation-reduction process (GO:0055114, BP) were the most enriched items at both 6 and 12 h (Figure 6).

### Kyoto Encyclopedia of Genes and Genomes Analysis

**FIGURE 7:**
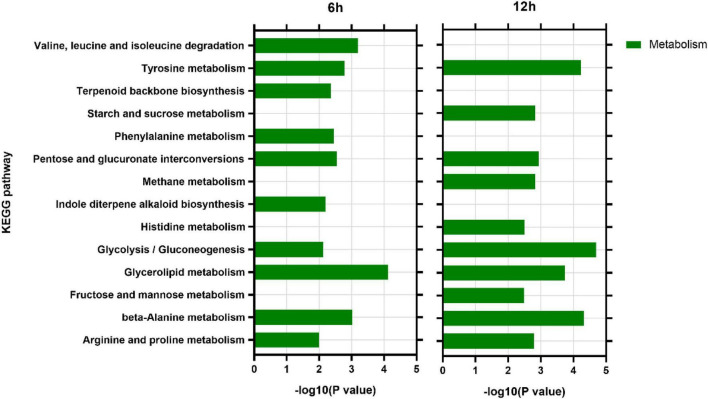
Analysis of main KEGG pathways of differentially expressed genes.

KEGG analysis showed that at 6 h, DEGs were enriched in 71 KEGG pathways, among which 59 (83.10%) were metabolism pathways. At 12 h, DEGs were enriched into 68 KEGG pathways, of which 56 (83.58%) were metabolic pathways (Supplementary Table 3). Among the top 10 enriched pathways at each time point, six pathways including tyrosine metabolism (ko00280), arginine and proline metabolism (ko00330), pentose and glucuronate interconversions (ko00040), glycolysis/gluconeogenesis (ko0 0010), and glycerolipid metabolism (ko00561) and beta-alanine metabolism (ko00410) were the most enriched at both time points (Figure 7).

### Differentially Expressed Genes Related to Membrane

**FIGURE 8:**
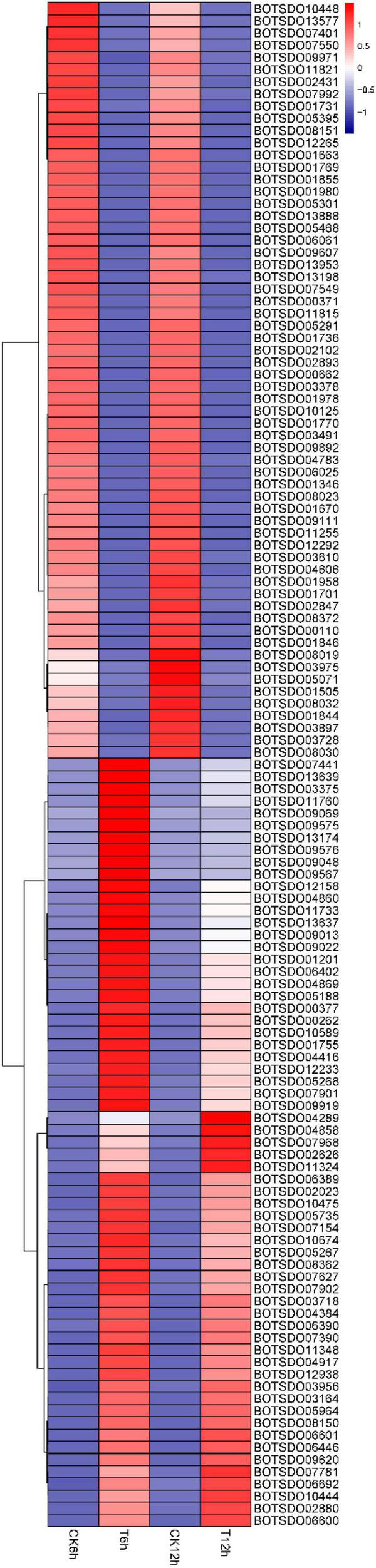
DEGs involved in GO term membrane.

Cell membrane plays an essential role in maintaining integrated cell structure, cell substance synthesis and exchange, signal transduction, protein synthesis, and other cellular activities. GO analysis revealed a total of 256 DEGs were enriched into the GO term membrane (GO:0016020). Among these genes, 125 DEGs, including alternate oxidase (BOTSD010674), cytochrome P450 (BOTSD002023), GDSL-Type lipase (BOTSD000377), major facilitator superfamily (MFS) (BOTSD004416), and GPR1/FUN34/YaaH (GFY) (BOTSD007968), were significantly differentially expressed at both 6- and 12-h time point (Figure 8), highly responding to *S. plymuthica* treatment.

### Differentially Expressed Genes Related to Glycerolipid Metabolism

**FIGURE 9:**
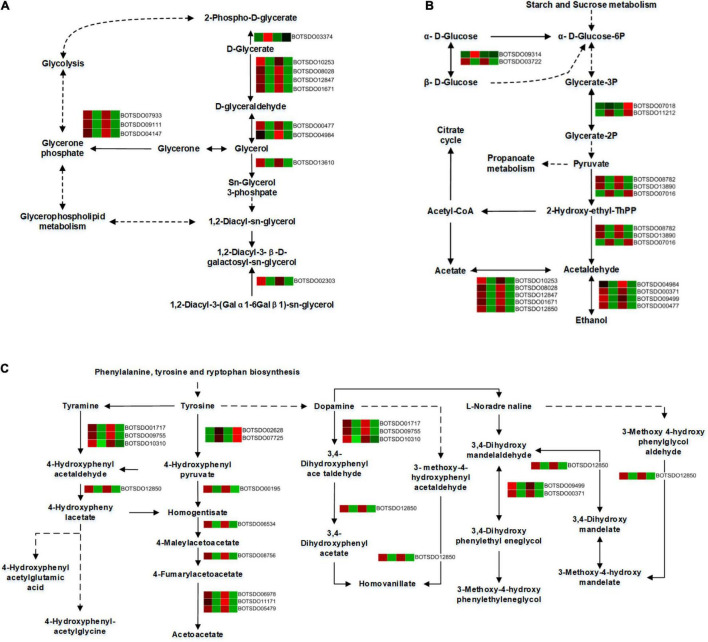
DEGs involved in glycerolipid metabolism **(A)**, glycolysis/gluconeogenesis **(B)**, and tyrosine metabolism **(C)** by KEGG analysis.

A total of 12 DEGs were involved in the glycerolipid metabolism. All the DEGs, including Aldo/keto reductase (BOTSDO04984), alcohol dehydrogenase (BOTSDO00477), and aldehyde dehydrogenase protein (BOTSDO10253, BOTSDO12847, BOTSDO01671), were significantly downregulated by *S. plymuthica* except glycerate kinase (BOTSDO03374) at both 6 and 12 h (Figure 9A), which revealed *S. plymuthica* slowed down the glycerolipid metabolism in *B. dothidea*.

### Differentially Expressed Genes Associated With Glycolysis/Gluconeogenesis

The glycolysis/gluconeogenesis pathway contained 16 DEGs, 12 of which were significantly downregulated responding to *S. plymuthica* stress. These genes include alcohol dehydrogenase superfamily zinc-containing (BOTSDO00371, BOTSDO00477, BOTSDO09499), aldehyde dehydrogenase protein (BOTSDO10253, BOTSDO12847, BOTSDO12850, BOTSDO01671), Aldo/keto reductase (BOTSDO04984), and DNA repair protein (BOTSDO03722). Except that, 4 DEGs such as phosphoglycerate mutase (BOTSDO07018, BOTSDO11212), thiamine pyrophosphate enzyme (BOTSDO07016), and hypothetical protein CC84DRAFT_425836 (BOTSDO09314) were upregulated responding to *S. plymuthica* (Figure 9B).

### Differentially Expressed Genes Related to Tyrosine Metabolism

In the tyrosine metabolism pathway, a total of 14 DEGs were screened at both time points. Twelve DEGs consisting of copper amine oxidase (BOTSDO01717, BOTSDO10310, BOTSDO09755), fumarylacetoacetase (BOTSDO11171, BOTSDO06978, BOTSDO05479), alcohol dehydrogenase (BOTSDO00371, BOTSDO09499), and aldehyde dehydrogenase (BOTSDO12850) were significantly regulated compared to the responding control at 6 and 12 h. In contrast, the two DEGs, aspartate aminotransferase (BOTSDO07725) and the hypothetical protein MPH_00577 (BOTSDO02628), were upregulated at both time points (Figure 9C).

### Quantitative RT-PCR Validation of RNA-Sequencing Data

**FIGURE 10:**
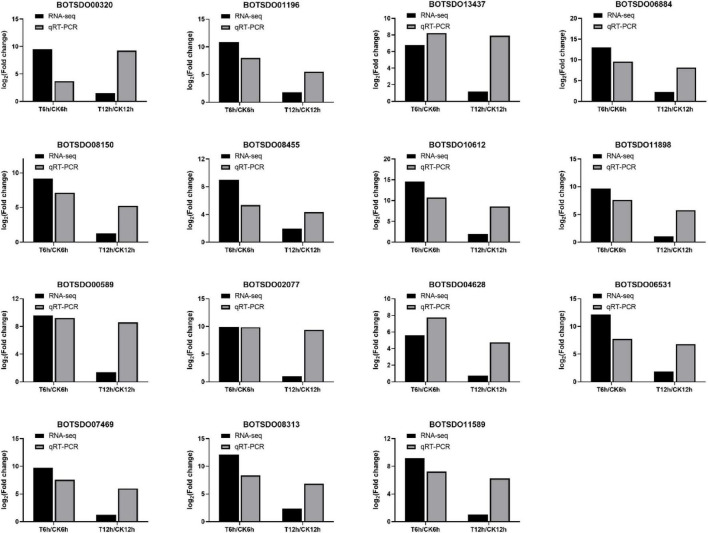
qRT-PCR validation of 15 randomly selected differentially expressed genes identified by Illumina high throughput RNA-sequencing.

To validate the RNA-seq results, we performed qRT-PCR on 15 genes differentially expressed between control and *S. plymuthica* treated mycelia (Figure 10). RNA-seq results showed that the 15 genes were upregulated by *S. plymuthica*, and qRT-PCR also revealed that those genes were upregulated by *S. plymuthica*. Though the gene change fold was not precisely the same, the changing trend was the same, which showed the qRT-PCR results were consistent with RNA-Seq results, thereby confirming the reliability of RNA-Seq data.

## Discussion

Apple ring rot is spreading across the orchards across China, seriously threatening the healthy development of apple fruit. In our preliminary study, we isolated an endophytic bacterium *S. plymuthica* from Chinese leek rhizome. The present study demonstrate that the *S. plymuthica* significantly suppresses the growth of *B. dothidea* and inhibits the apple ring rot on postharvest fruits. In addition, *S. plymuthica* also improves the fruit’s quality. Therefore, the study provides a possible way to control the apple ring rot of postharvest fruits.

In the present study, the endophytic bacterial *S. plymuthica* exerts an antifungal activity of 52.5% against *B. dothidea* growth and suppresses the apple ring rot by 84.64%. Besides *S. plymuthica*, several species in *Serratia* sp. show potent antifungal activity. *Serratia* sp. isolated from *Bacopa monnieri* has effective inhibition against *Pythium myriotylum*, *Rhizoctonia solani*, *Sclerotium rolfsii*, *Phytophthora infestans*, and *Fusarium oxysporum* (Jimtha et al., 2017). *S. marcescens* significantly suppresses the growth of *Rhizoctonia solani*, *Fusarium oxysporum* (Do et al., 2017), and *Didymella applanata* (Duzhak et al., 2012). *S. rubidaea* exhibits high suppression against *Fusarium oxysporum* and *Colletotrichum gloeosporioides* (Nalini and Parthasarathi, 2014). *S. proteamaculans* 336x significantly suppresses the growth of *Gaeumannomyces graminis* var. *tritici*, and reduces the occurrence of wheat take-all (Wang et al., 2014). Together with all these previous studies, the present study suggests *Serratia* sp. is a good resource for developing antagonistic bacteria to prevent and control various plant pathogens.

To preliminary explore the molecular mechanism of the inhibition of *S. plymuthica* against *B. dothidea*, we screen 777 and 890 DEGs using RNA-seq from the comparisons of the control mycelia and the *S. plymuthica-*treated mycelia at 6 and 12 h, respectively. Among these DEGs, alternate oxidase (AOX), cytochrome P450 (CYP450), GDSL-Type lipase, MFS, and GFY, enriched into GO term membrane, highly responded to *S. plymuthica* at both time points. These membrane-related proteins can participate in the metabolism of many exogenous substances, including drugs and environmental stress, which is significant for maintaining normal cell growth and development. Alternative oxidase regulates the growth, development, and resistance to oxidative stress of *Sclerotinia sclerotiorum* (Xu et al., 2012). In addition, alternative oxidase gene improves the resistance of *Monascus ruber* to stressful conditions such as H_2_O_2_, high temperature, and alkaline buffer (Shao et al., 2017), and the resistance of *Vibrio fischeri* to nitric oxide, and enhances bacterial fitness and survival under stressful environmental conditions (Dunn, 2018). Cytochrome P450 gene is required for the biosynthesis of the trichothecene toxin harzianum A in *Trichoderma* (Cardoza et al., 2019) and promotes cell survival of the sugarcane smut fungus *Sporisorium scitamineum* under oxidative stress (Cai et al., 2021). The GDSL lipase exhibits high tolerance against detergents and metal ions in *Geobacillus thermocatenulatus* (Jo et al., 2021). MFS gene mfs1 or mfs2 reduce the susceptibility to aminoglycosides, quinolones, and paraquat in *Pseudomonas aeruginosa* (Dulyayangkul et al., 2021) and play an essential role in resistance to toxicity, oxidants, and fungicides in *Alternaria alternata* (Lin et al., 2018). GFY gene *AcpA* in fungus *Aspergillus nidulans* (Robellet et al., 2008) is an essential component of mediated acetate transport.

In the present study, more than 83.00% of the KEGG pathway belong to the metabolic pathway. Glycerolipid metabolism, glycolysis/gluconeogenesis, and tyrosine metabolism are the most enriched pathways at both time points. We believe the inhibition of *S. plymuthica* against *B. dothidea* is closely related to these pathways. Previous studies show that DEGs such as alcohol dehydrogenase, aldehyde dehydrogenase gene, DNA repair protein, aspartate aminotransferases, and aldo/keto reductase contained in these pathways played a vital role in fungi. For instance, *Synechocystis* alcohol dehydrogenase sysr1 considerably increases tolerance in salt stress conditions (Yi et al., 2017), and *Botrytis cinerea* alcohol dehydrogenase *BcADH1* is required for fungal development, environmental adaptation, and its ability for full pathogenicity (DafaAlla et al., 2021). *ald1*, an aldehyde dehydrogenase gene in the fungus *Tricholoma vaccinum*, increases its tolerance to ethanol stress (Asiimwe et al., 2012). *Saccharomyces cerevisiae* DNA repair gene RAD6 is necessary to repair damaged DNA in stress situations (Madura et al., 1990). *Beauveria bassiana* aldo-keto reductase, Bbakr1, is involved in osmotic and salt stressors and oxidative and heavy metal (chromium) stress (Wang et al., 2018). Aspartate aminotransferase gene governs aspartate biosynthesis and nitrogen distribution in *Mycobacterium tuberculosis* (Jansen et al., 2020) and activates TOR complex 1 in fission yeast, improving cell growth and proliferation in response to nutritional signals (Reidman et al., 2019).

Good measures should not only control postharvest diseases on fruit but also maintain fruit quality. Previous studies show that alginate oligosaccharide (Bose et al., 2019), brassinosteroids (Furio et al., 2019), acibenzolar-S-methyl (Li et al., 2020), ozone (Luo et al., 2019), and integration of ultraviolet-C with antagonistic yeast *Candida tropicalis* (Ou et al., 2016) strongly inhibit the disease occurrences on postharvest fruits, and also significantly improve these fruit qualities. In the present study, *S. plymuthica* significantly suppresses the occurrence of apple ring rot on postharvest fruit, reduces the TA content, increases the SS content, TSS/TA ratio, and SS/TA ratio, and maintains apple fruit’s firmness. Thus, these previous studies and *S. plymuthica* used in the present study fulfill the criteria of good measure to control postharvest disease, which can kill two birds with one stone. In addition, the *S. plymuthica* is isolated from Chinese leek, a popular vegetable in China. Therefore, using the endophytic bacterium *S. plymuthica* to control apple ring rot on postharvest fruit is safer for humans and animals and more environment-friendly.

From the RNA analysis results, we deduce that *S. plymuthica* damages the membrane of *B. dothidea*, resulting in membrane-related gene expression altered and causing the oxidation-reduction imbalance, eventually leading to its amino acid, carbohydrate, and lipid, energy, and other metabolic disorders, which eventually affect the healthy growth of *B. dothidea*.

## Conclusion

The present study demonstrates that the endophytic bacterium *S. plymuthica* isolated from Chinese leek significantly suppresses the growth of *B. dothidea*, and exerts a remarkable inhibition against apple ring rot on postharvest fruits. *S. plymuthica* reduces the TA content, increases the SS content, TSS/TA ratio, and SS/TA ratio, and maintains apple fruit’s firmness. Furthermore, transcriptome analysis shows *S. plymuthica* mainly alters genes related to the metabolism pathway of *B. dothidea.* Thus, the study provides a possible way to control apple ring rot disease and lay a solid basis for further exploring the molecular mechanism of *S. plymuthica* inhibition against *B. dothidea*.

## Data Availability Statement

The RNA-seq data were deposited at the National Center for Biotechnology Information under accession number PRJNA727902.

## Author Contributions

YH contributed to the conception of the study and revised the manuscript. MS wrote the manuscript, performed the experiment, and collected the data. JPL and JHL experimented and collected the data. All authors contributed to the article and approved the submitted version.

## Conflict of Interest

The authors declare that the research was conducted in the absence of any commercial or financial relationships that could be construed as a potential conflict of interest. The reviewer PL declared a shared affiliation with the authors to the handling editor at the time of review.

## Publisher’s Note

All claims expressed in this article are solely those of the authors and do not necessarily represent those of their affiliated organizations, or those of the publisher, the editors and the reviewers. Any product that may be evaluated in this article, or claim that may be made by its manufacturer, is not guaranteed or endorsed by the publisher.
